# Hepatitis C virus core protein triggers abnormal porphyrin metabolism in human hepatocellular carcinoma cells

**DOI:** 10.1371/journal.pone.0198345

**Published:** 2018-06-01

**Authors:** Takafumi Nakano, Kyoji Moriya, Kazuhiko Koike, Toshiharu Horie

**Affiliations:** 1 Biopharmaceutics and Molecular Toxicology Unit, Faculty of Pharmaceutical Sciences, Teikyo Heisei University, Nakano-ku, Tokyo, Japan; 2 Department of Internal Medicine, Graduate School of Medicine, The University of Tokyo, Bunkyo-ku, Tokyo, Japan; Nihon University School of Medicine, JAPAN

## Abstract

Porphyria cutanea tarda (PCT), the most common of the human porphyrias, arises from a deficiency of uroporphyrinogen decarboxylase. Studies have shown a high prevalence of hepatitis C virus (HCV) infection in patients with PCT. While these observations implicate HCV infection as a risk factor for PCT pathogenesis, the mechanism of interaction between the virus and porphyrin metabolism is unknown. This study aimed to assess the effect of HCV core protein on intracellular porphyrin metabolism to elucidate the link between HCV infection and PCT. The accumulation and excretion of porphyrins after treatment with 5-aminolevulinic acid, a porphyrin precursor, were compared between cells stably expressing HCV core protein and controls. Cells expressing HCV core protein had lower amounts of intracellular protoporphyrin IX and heme and had higher amounts of excreted coproporphyrin III, the oxidized form of coproporphyrinogen III, compared with controls. These observations suggest that HCV core protein affects porphyrin metabolism and facilitates the export of excess coproporphyrinogen III and/or coproporphyrin III, possibly via porphyrin transporters. Real-time PCR analysis revealed that the presence of HCV core protein increased the mRNA expression of porphyrin exporters ABCG2 and FLVCR1. Western blot analysis showed a higher expression level of FLVCR1, but not ABCG2, as well as a higher expression level of mature ALAS1, which is the rate-limiting enzyme in the heme synthesis pathway, in HCV core protein-expressing cells compared with controls. The data indicate that HCV core protein induced abnormal intracellular porphyrin metabolism, with an over-excretion of coproporphyrin III. These findings may partially account for the susceptibility of HCV-infected individuals to PCT development.

## Introduction

Chronic hepatitis caused by the hepatitis C virus (HCV) is a major health problem affecting 120–200 million people worldwide. Patients with long-lasting HCV infection are at major risk of developing hepatocellular carcinoma. In addition to causing liver diseases, chronic hepatitis C has a broad spectrum of extrahepatic manifestations [1, 2], including cryoglobulinemia, membranoproliferative glomerulonephritis, and porphyria cutanea tarda (PCT). Porphyrias are rare disorders of porphyrin metabolism that result in porphyrin accumulation. PCT, the most common type of porphyria, is associated with a defect in uroporphyrinogen decarboxylase (UROD), the fifth enzyme in the heme biosynthetic pathway in the liver. Extrinsic factors, such as alcohol intake and estrogen therapy, are known to trigger PCT [3]. The possible role of HCV infection in PCT pathogenesis has recently been postulated based on the high prevalence of HCV infection in patients with PCT [4]. Although the association between PCT and HCV infection is now strongly established, the mechanism that links chronic HCV infection to PCT pathogenesis remains unknown.

The HCV genome includes a single open reading frame that encodes a precursor polyprotein. This large polyprotein undergoes host and viral protease-mediated posttranslational modification to generate at least ten smaller proteins, including three structural proteins (the core protein and two envelope proteins E1 and E2), viroporin p7 and six non-structural proteins (NS2, NS3, NS4A, NS4B, NS5A and NS5B) [5]. These proteins not only participate in viral replication, but also influence host cellular functions. HCV core protein, which is derived from the N-terminus of the precursor polyprotein, localizes to endoplasmic reticulum, fat droplets as well as mitochondria. It produces multiple changes in gene transcription, signal transduction, immune presentation, and cell-cycle regulation [6]. In particular, the HCV core protein has a functional effect on mitochondria and causes an increase in host ROS production, lipid peroxidation, and mitochondrial Ca^2+^ uptake, and decreases GSH and NADPH concentrations and mitochondrial complex I activities [7–10].

We recently reported that HCV core protein up-regulates iron uptake into the mitochondria and exacerbates oxidative stress and hepatic toxicity [11]. As heme synthesis is a major route of iron use in hepatocytes, the HCV core protein likely also affects heme synthesis (i.e., porphyrin metabolism). The present study aimed to determine the effect of HCV core protein on the heme biosynthetic pathway in human hepatocellular carcinoma cells to better understand the link between HCV infection and PCT.

## Materials and methods

### Chemicals and reagents

5-aminolevulinic acid (ALA) was obtained from Wako (Osaka, Japan). Protoporphyrin IX, coproporphyrin III, and porphyrin acid chromatographic marker were from Frontier Scientific (Logan, UT, USA). ReverTra Ace^®^ qPCR RT kit was obtained from Toyobo (Osaka, Japan). TRIzol Reagent and Fast SYBR Green Master mix were purchased from Thermo Fisher Scientific (Waltham, MA, USA). Anti-ALAS1 antibody (EPR10247), anti-FLVCR antibody (ab70838), anti-BCRP/ABCG2 antibody (EPR2099 (2)), and anti-COX IV antibody (20E8C12) were purchased from Abcam (Cambridge, MA, USA). Anti-HCV core protein antibody was purchased from Anogen (Mississauga, Canada). Anti-ABCB6 antibody was purchased from Proteintech Group (Chicago, IL, USA). Anti-β-actin monoclonal antibody was purchased from MBL Co., Ltd. (Nagoya, Japan). Unless otherwise noted, all other chemicals and solvents were of an analytical grade.

### Cell culture

HepG2 cell lines expressing the HCV core protein (Hep39, Hep39b) and a control HepG2 line (Hepswx) carrying the empty vector were described previously [12–14]. Hepswx, Hep39, and Hep39b cells were cultured in Dulbecco’s modified Eagle’s medium (Sigma-Aldrich) containing glucose (4,500 mg/L), penicillin (100 U/ml), streptomycin (100 μg/ml), amphotericin B (0.25 μg/ml), G418 (0.4 mg/ml), and 10% fetal bovine serum at 37°C in a humidified atmosphere of 5% CO_2_ and 95% air.

### Extraction of heme and porphyrins

Cells were seeded into six-well culture plates at a density of 2.0 × 10^5^ cells/cm^2^ and, after 24 h, were exposed to various concentrations of ALA for 1–3 days. For extracellular uroporphyrin extraction, 900 μl of medium was collected and supplemented with 2 μl of 0.5 M EDTA (pH 8.0). A 100-μl aliquot of 100% (w/v) trichloroacetic acid was added. The mixture was centrifuged at 16,500 × *g* for 10 min, and the resulting supernatant was collected. To extract extracellular coproporphyrin III and protoporphyrin IX, 200 μl of medium was collected and supplemented with 2.4 μl of 0.5 M EDTA (pH 8.0), and 1 ml of acetone–37% (mass/mass) HCl (39:1 by volume) was added. The mixture was centrifuged at 16,500 × *g* for 10 min, and the resulting supernatant was collected. For intracellular porphyrin extraction, cells were washed twice with phosphate-buffered saline, detached using a cell scraper, collected by centrifugation (300 × *g*, 5 min), and re-suspended in 1 ml of ethyl acetate–acetic acid (4:1, by volume) supplemented with 2 μl of 0.5 M EDTA. The cell suspension was protected from light, incubated overnight, and then centrifuged at 16,500 × *g* for 10 min. The supernatant was collected for HPLC analysis, and the pellet was used for protein assays. The pellet was dissolved in 0.5 ml of 1 N NaOH and neutralized with 0.5 ml of 1 N HCl. The protein concentration of the resulting cell lysate was then determined using the BCA Protein Assay Reagent Kit (Pierce Chemical Co., Rockford IL). For intracellular heme extraction, the collected cells were re-suspended in 1 ml of acetone–37% (mass/mass) HCl–H_2_O (39:1:8, by volume). The remainder of the procedure was the same as that for the porphyrin extraction described above.

### Analytical method for HPLC analysis of heme and porphyrins

All analyses were performed using a Prominence HPLC system (Shimadzu, Kyoto, Japan) and ZORBAX Eclipse XDB-C18 column (4.6 × 150 mm, Agilent Technologies, Santa Clara, CA, USA). The chromatographic conditions employed for analysis of porphyrins and hemes were as follows: Mobile phase A, 15% acetonitrile:1 M ammonium acetate (pH 5.15); mobile phase B, 90% acetonitrile:50 mM ammonium acetate (pH 5.15). A linear gradient was programmed from 100% mobile phase A to 40% mobile phase B over 12 min, then to 100% mobile phase B over 1 min, where it was held for 7 min, and finally returned to the initial conditions using a 1-min linear gradient followed by re-equilibration for 4 min. The flow rate was 1.0 ml/min. Heme absorbance was measured at 400 nm, and porphyrin fluorescence was detected at 400 nm (excitation) and 630 nm (emission).

### RNA preparation and quantitative real-time RT-PCR

Cells were treated with or without 0.5 mM ALA for 3 days. After treatment, total RNA was extracted using TRIzol according to the manufacturer’s instructions. The concentration and quality of RNA were analyzed using a NanoDrop Lite spectrophotometer (Thermo Fisher Scientific, Waltham, MA, USA). Reverse transcription was performed using the ReverTra AceqPCR RT kit (Toyobo) according to the manufacturer’s instructions. The expression of 5-aminolevulinate synthase 1 (ALAS1), 5-aminoluvurinate dehydratase (ALAD), hydroxymethylbilane synthase (HMBS), uroporphyrinogen III synthase (UROS), uroporphyrinogen decarboxylase (UROD), coproporphyrinogen oxidase (CPOX), protoporphyrinogen oxidase (PPOX), ferrochelatase (FECH), heme oxygenase 1 (HO-1), ABCB6, ABCG2, FLVCR1, and GAPDH mRNAs were determined using the StepOnePlus real-time PCR system (Applied Biosystems) with the Fast SYBR Green Master Mix (Applied Biosystems) and specific primer sets (S1 Table). Expression levels of target genes were normalized to those of GAPDH.

### SDS-PAGE and immunoblot analysis

To prepare the samples for immunoblot analysis, cells were lysed using the EzRIPA lysis kit (ATTO, Tokyo, Japan), and mitochondria were isolated using the Mitochondria Isolation kit (BioChain Institute Inc., Gibbstown, NJ, USA), both according to the manufacturer’s instructions. The protein concentration was determined using the Pierce BCA Protein Assay Reagent kit. Proteins were lysed in Laemmli sample buffer, subjected to SDS-polyacrylamide gel electrophoresis, and transferred onto an Immobilon-P Transfer Membrane filter (Millipore Co., Bedford, MA, USA). The membrane was blocked for 1 h at room temperature with Tris-buffered saline containing 0.1% Tween 20 (TTBS) and 3% skim milk. Blocked membranes were then probed at room temperature for 1 h or at 4°C overnight with primary antibodies diluted in TTBS containing 0.1% skim milk. The membrane was subsequently incubated for 1 h at room temperature with horseradish peroxidase-conjugated secondary antibodies diluted in TTBS containing 0.1% skim milk. Bound antibodies were detected using an LAS-4000 Luminescent Image Analyzer (GE Healthcare Inc., St. Louis, MO, USA) and an enhanced chemiluminescence detection system (GE Healthcare Inc.)

### Statistical analysis

All data are represented as the mean ± standard error (SE). Data were statistically analyzed using a one-way analysis of variance followed by Dunnett’s post-hoc test, as appropriate. For comparisons between two groups, a two-tailed paired Student’s *t*-test was adopted. Differences between means at the level of *p* < 0.05 were considered significant.

## Results

### The effect of HCV core protein on the intracellular heme synthesis pathway in 5-aminolevlinic acid-treated HepG2 cells

**Fig 1:**
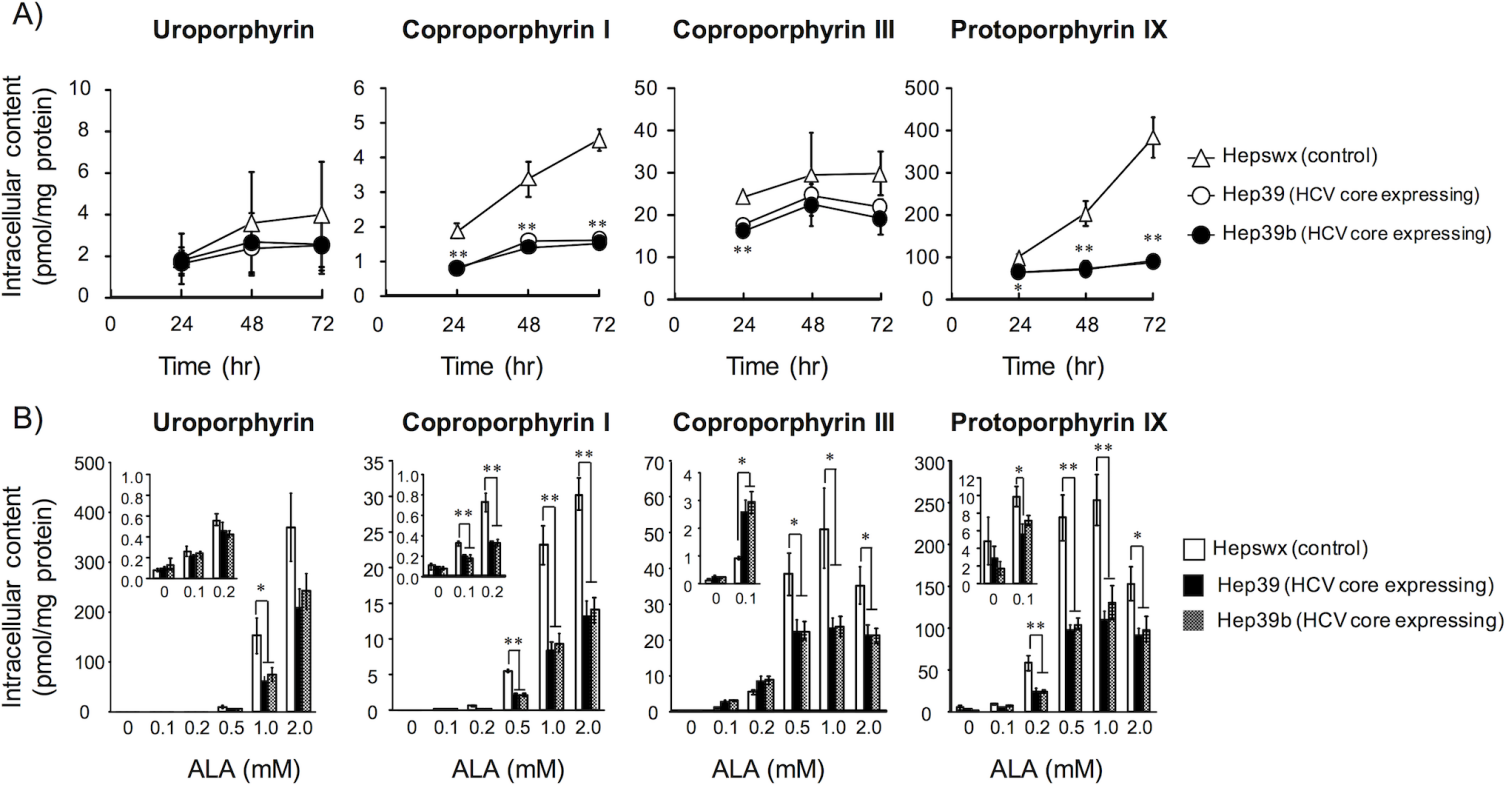
The effect of HCV core protein on intracellular porphyrin accumulation. A) Time profile of intracellular porphyrin accumulation in ALA-treated cells. Control (Hepswx, Δ) and HCV core protein-expressing (Hep39, ◯; Hep39b, ●) cells were seeded into six-well culture plates at a density of 2.0 × 10^5^ cells/cm^2^; after 24 h, the cells were incubated with 0.5 mM ALA for the indicated times. Intracellular porphyrins were extracted as described in the Materials and methods. B) Relationship between ALA concentration in the incubation medium and intracellular porphyrin accumulation. Cells were seeded into six-well culture plates at a density of 2.0 × 10^5^ cells/cm^2^; after 24 h, the cells were incubated with the indicated concentration of ALA for 3 days, and the intracellular porphyrins were extracted. Data are presented as the mean ± SE of 3–5 independent experiments. **p < 0.01 and *p < 0.05, significantly different from the respective control.

Cells have two uroporphyrin isomers, uroporphyrin I and uroporphyrin III. We could not achieve complete separation of the peaks of these isomers under the HPLC conditions used in this study. Therefore, uroporphyrin was assessed as the sum of uroporphyrin I and uroporphyrin III. To determine the effect of HCV core protein on intracellular heme and porphyrin metabolism, the accumulated porphyrins in ALA-treated cells stably expressing HCV core protein (Hep39 and Hep39b cells) and vector control cells (Hepswx cells; control) were evaluated by HPLC analysis. ALA treatment did not affect the cell viability in HCV core-expressing and control cells at least up to 1 mM (S1 Fig). The time courses of porphyrin accumulation in these cells are shown in Fig 1A. Following ALA treatment, the content of intracellular coproporphyrin I and protoporphyrin IX increased in a time-dependent manner for 3 days in control cells but in an obscure manner in HCV core protein-expressing cells. The ALA-induced accumulations of uroporphyrin and coproporphyrin III, however, were not clearly time-dependent in either cell type.

**Fig 2:**
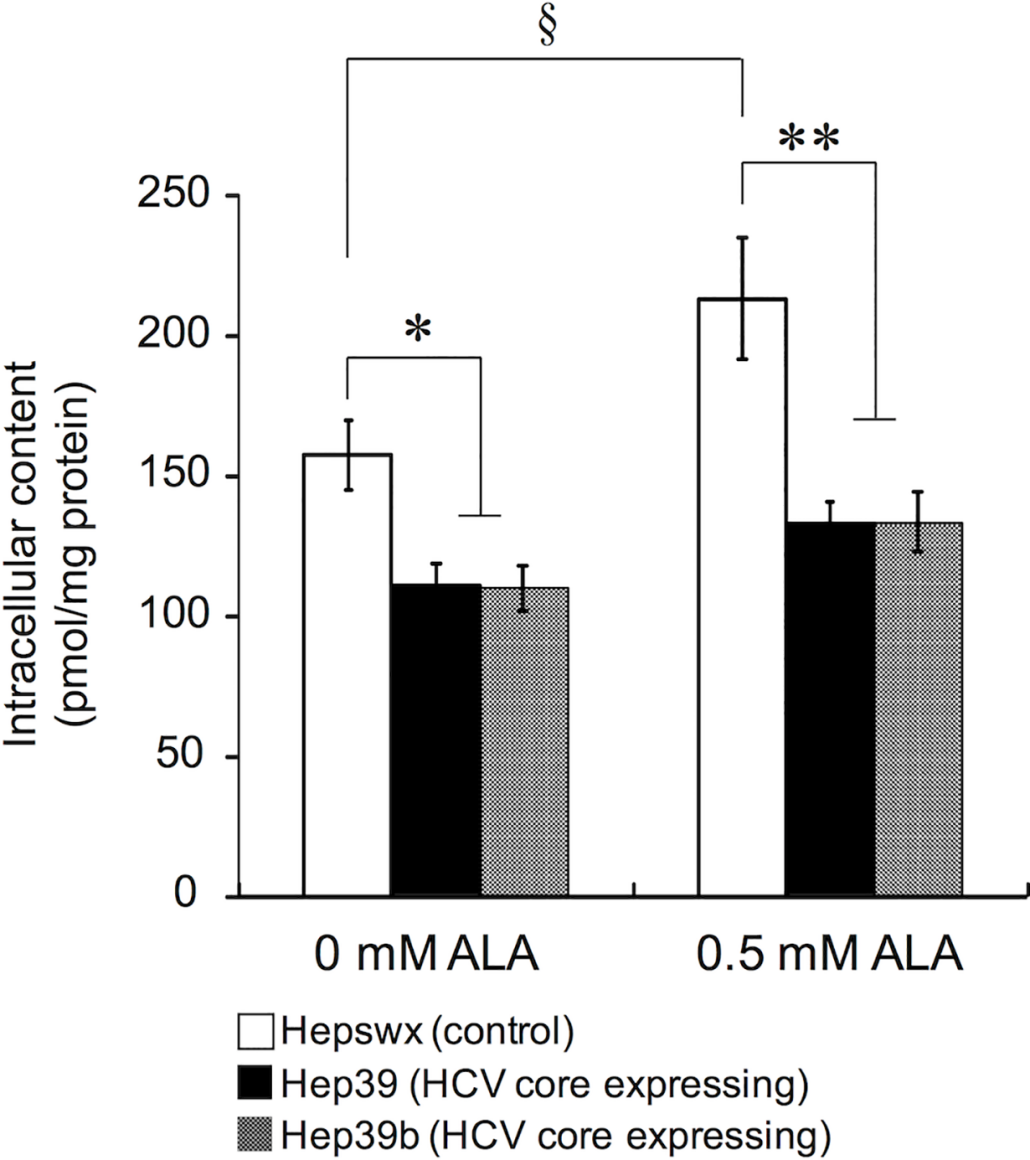
Intracellular heme content in control and HCV core protein-expressing cells. Cells were seeded into six-well culture plates at a density of 2.0 × 10^5^ cells/cm^2^; after 24 h, the cells were treated with or without 0.5 mM ALA for 3 days. The intracellular heme was extracted as described in the Materials and methods. Data are presented as the mean ± SE of four independent experiments. **p < 0.01, *p < 0.05, and ^§^*p* < 0.05, significantly different from the respective control.

After treatment with ALA for 3 days, accumulated uroporphyrin and copropophyrin I increased in a concentration-dependent manner over the full range of tested ALA concentrations. Accumulated copropophyrin III and protoporphyron IX increased in a concentration-dependent manner up to 0.5 mM of ALA; however, at ALA concentrations of 1.0 mM and 2.0 mM, the content of these porphyrins decreased (Fig 1B). HCV core protein expression decreased the intracellular accumulation of protoporphyrin IX and coproporphyrin I in the tested dose range of ALA. The intracellular content of heme, which is the final product of porphyrin metabolism, was lower in HCV core protein-expressing cells than in control cells, both with and without ALA treatment (Fig 2). In contrast, extracellular heme was not detected. These data indicate that HCV core protein expression affects intracellular heme synthesis.

### The effect of HCV core protein on porphyrin excretion

**Fig 3:**
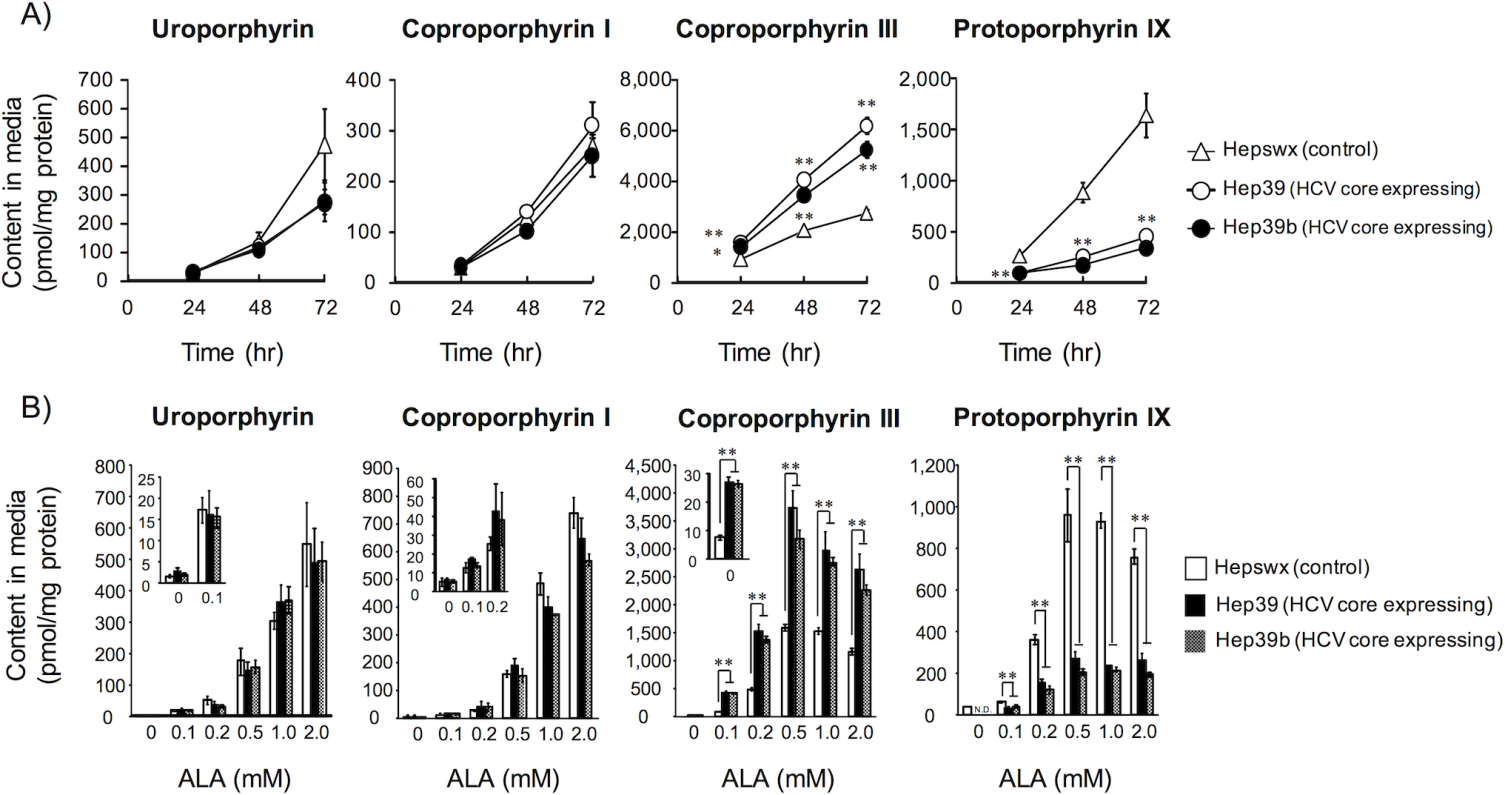
The effect of HCV core protein on porphyrin excretion. A) Time profile of extracellular porphyrin accumulation in ALA-treated cells. Control (Hepswx, Δ) and HCV core protein-expressing (Hep39, ◯; Hep39b, ●) cells were seeded into six-well culture plates at a density of 2.0 × 10^5^ cells/cm^2^; after 24 h, the cells were incubated with 0.5 mM ALA for the indicated times. Porphyrins in the medium were extracted as described in the Materials and methods. B) Relationship between ALA concentration in the incubation medium and the intracellular porphyrin accumulation. Cells were seeded into six-well culture plates at a density of 2.0 × 10^5^ cells/cm^2^; after 24 h, the cells were incubated with the indicated concentration of ALA for 3 days. Porphyrins in the medium were extracted. Data are presented as the mean ± SE of 3–6 independent experiments. **p < 0.01 and *p < 0.05, significantly different from the respective control.

To investigate porphyrin excretion from cells, the porphyrin content of the medium was assessed. Time courses of porphyrin excretion are shown in Fig 3A. All porphyrins were excreted in a time-dependent manner over the 3 days that these experiments lasted. The expression of HCV core protein decreased the protoporphyrin IX excretion but increased the coproporphyrin III excretion and had no effect on the uroporphyrin or coproporphyrin I excretion in ALA-treated cells. After treatment with ALA for 3 days, the excretion levels of both uroporphyrin and copropophyrin I increased in a concentration-dependent manner over the entire range of tested ALA concentrations. Although excretion levels of copropophyrin III and protoporphyron IX increased in a concentration-dependent manner up to 0.5 mM of ALA, higher ALA concentrations of 1.0 mM and 2.0 mM led to a decrease in the excretion levels of these porphyrins (Fig 3B). Intracellular coproporphyrin III levels were lower and excreted coproporphyrin III levels were higher in HCV core protein-expressing cells as compared with control cells (Figs 1 and 3). These data suggest that intracellular coproporphyrin III is actively transported out of ALA-treated cells that are expressing the HCV core protein. To exclude the possibility that the effect of HCV core is specific for HepG2 cells, the effect of HCV core protein on porphyrin metabolism in Huh-7 cells (another human hepatoma cell line) were evaluated. As a result, ALA-treated HCV core protein stably expressing Huh-7 cells showed similar porphyrin pattern (the increase in coproporphyrin III excretion and the decrease in protoporphyrin IX accumulation) (S2 Fig).

### The effect of HCV core protein on the mRNA and protein expression of enzymes in the heme synthesis pathway

**Fig 4:**
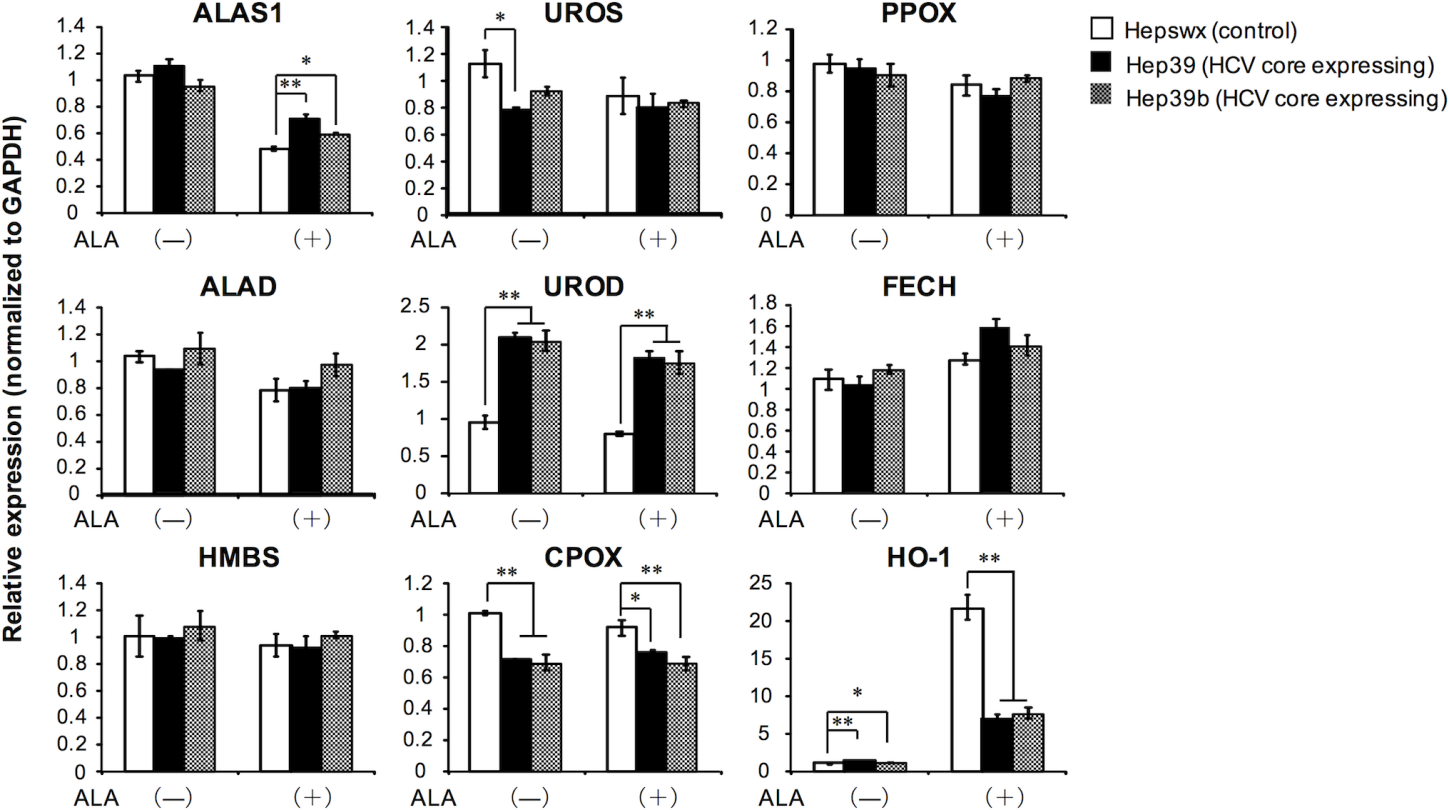
mRNA expression of enzymes involved in heme synthesis. Cells were treated with or without 0.5 mM ALA for 3 days before being harvested, and total RNA was then prepared from the cells. mRNA levels of enzymes involved in heme synthesis were quantified by real-time RT-PCR analysis. Data are presented as the mean ± SE of three independent experiments. **p < 0.01 and *p < 0.05, significantly different from the respective control.

**Fig 5:**
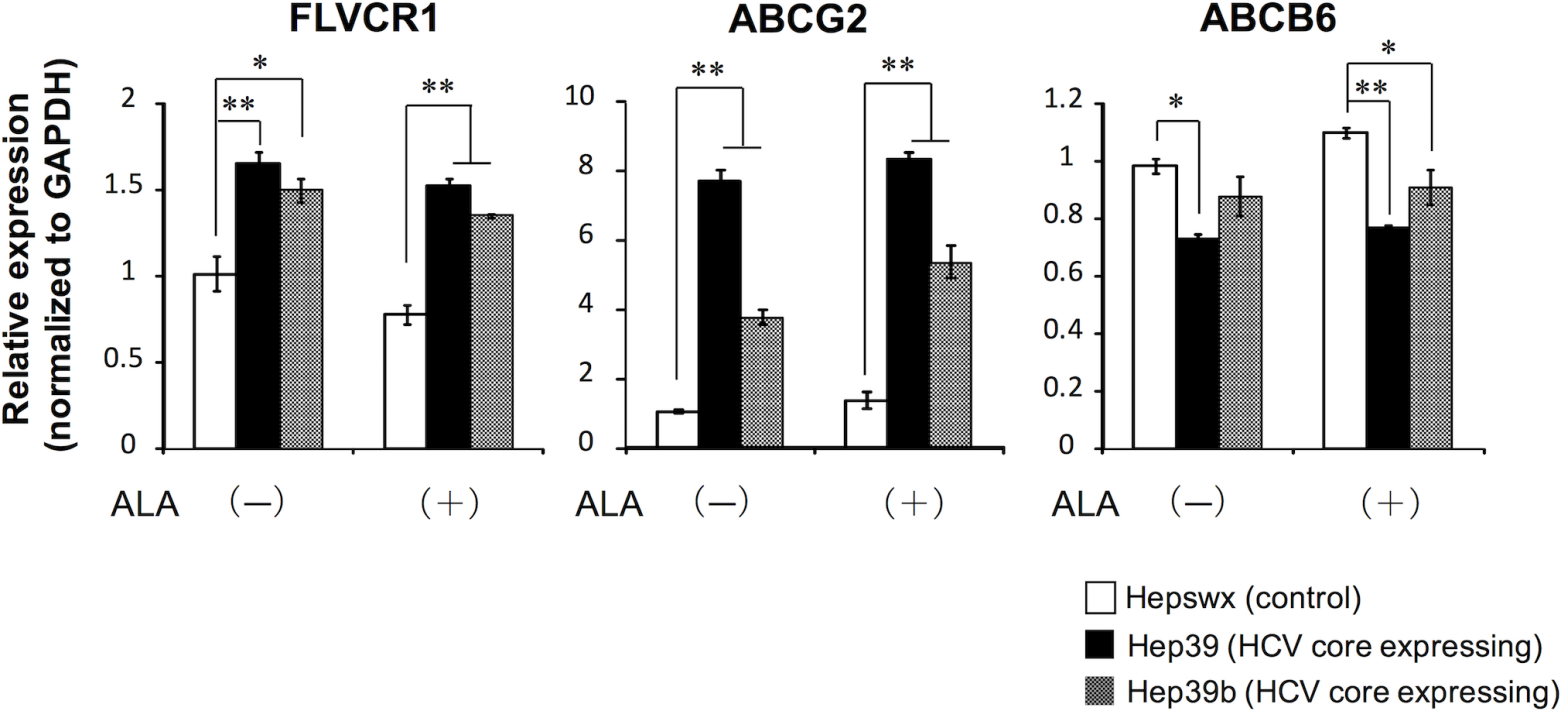
mRNA expression of heme/porphyrin transporters involved in heme synthesis. Cells were treated with or without 0.5 mM ALA for 3 days before being harvested, and total RNA was then prepared from the cells. mRNA levels of transporters involved in heme synthesis were quantified by real-time RT-PCR analysis. Data are presented as the mean ± SE of three independent experiments. **p < 0.01 and *p < 0.05, significantly different from the respective control.

To gain insight into the abnormal porphyrin metabolism in HCV core protein-expressing cells, the mRNA expressions of enzymes and transporters involved in heme synthesis were investigated. In HCV core protein-expressing cells, CPOX mRNA expression was significantly lower and UROD mRNA expression was significantly higher than those in control cells (Fig 4). Although the presence of HCV core protein increased the expression of heme oxygenase 1 (HO-1) mRNA, which encodes an enzyme that breaks down heme in the absence of ALA treatment, cells expressing the HCV core protein had lower levels of HO-1 mRNA induction following ALA treatment compared with control cells. Because heme is a potent inducer of HO-1, this result may be due to the lower intracellular heme content observed in these cells, as shown in Fig 2. Of the tested transporters involved in the heme synthesis pathway, ABCG2 and FLVCR1 had relatively high mRNA expression in HCV core protein-expressing cells compared with control cells, regardless of ALA treatment (Fig 5). In contrast, the mRNA expression of ABCB6, which is a mitochondrial porphyrin importer [15], was significantly lower in HCV core protein-expressing cells than that in control cells.

**Fig 6:**
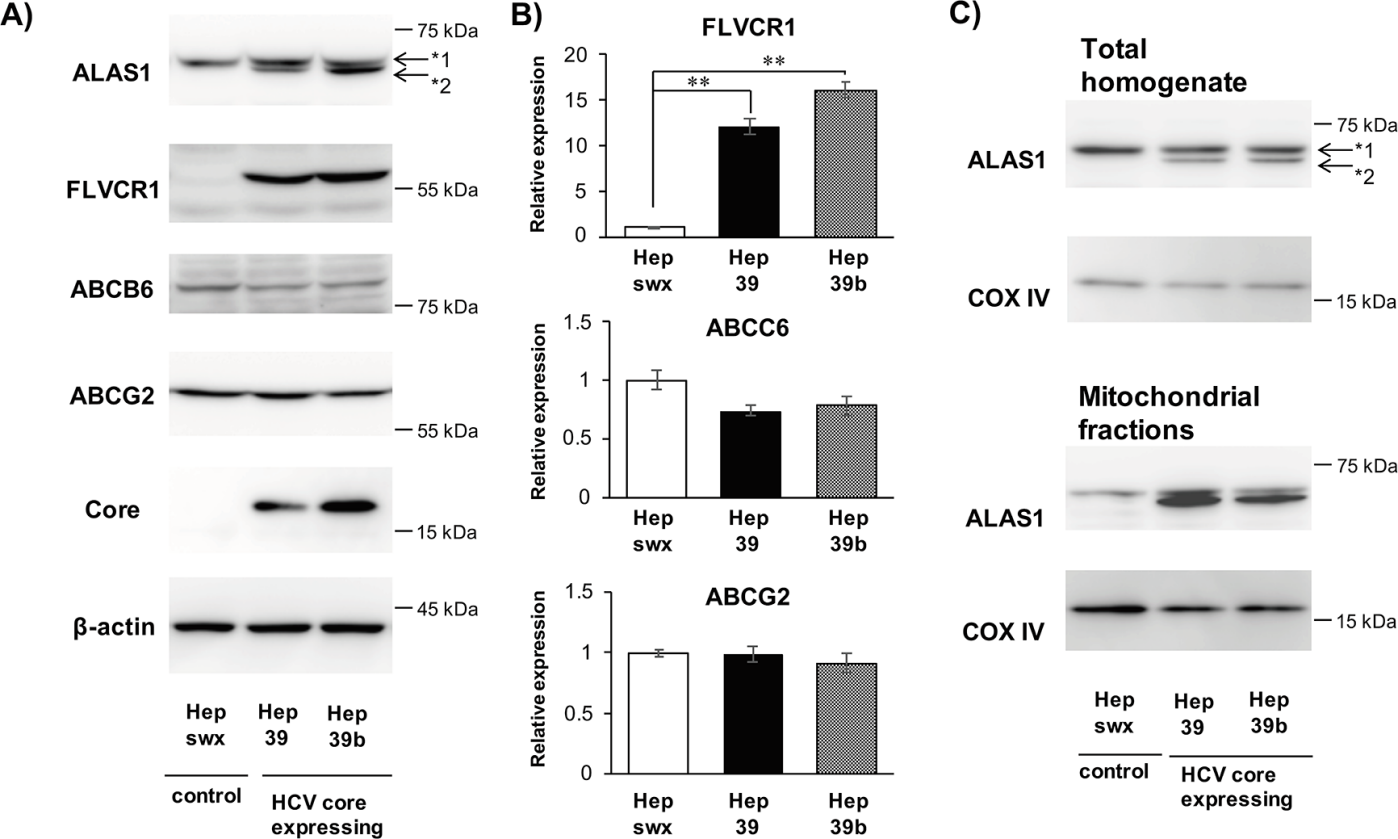
The protein expression level of ALAS1, FLVCR1, and ABCG2 in HCV core protein-expressing cells. A) Immunoblot analysis of whole cell lysates using primary antibodies against ALAS1, FLVCR1, ABCB6, ABCG2, and β-actin. B) Relative band density of FLVCR1, ABCB6 and ABCG2 in Fig 6A were quantified and displayed graphically. Data are presented as the mean ± SE of three independent experiments. **p < 0.01, significantly different from the respective control. C) Immunoblot analysis of total cell homogenates and mitochondrial fractions using primary antibodies against ALAS1 and COX IV, which is a mitochondrial marker. *1 indicates the precursor form of ALAS1. *2 indicates the mature form of mitochondrial ALAS1.

The protein expression of ALAS1, ABCG2, ABCB6 and FLVCR1 in total cell lysates was examined by immunoblot analysis. As shown in Fig 6A and 6B, FLVCR1 protein levels were significantly greater and ABCB6 protein levels slightly lower, but not significantly, in cells expressing the HCV core protein than in control cells as similar as the change in mRNA expression. In contrast, the ABCG2 protein level did not differ between tested cell types, despite the elevated mRNA level, suggesting post-transcriptional and/or translational modulation. As previously reported [16], we detected two different molecular weight forms of ALAS1, corresponding to the precursor form (higher MW) and mature form (lower MW). The level of mature ALAS1 protein was elevated in cells expressing HCV core protein and enriched in the mitochondrial fraction (Fig 6C). These results suggest that HCV core protein expression particularly increases the level of the mature form of the ALAS1 protein.

## Discussion

In the present study, HCV core protein significantly suppressed intracellular protoporphyrin IX and heme (Figs 1 and 2). These data indicate that HCV core protein disturbs the heme synthesis pathway downstream of the conversion or transport of coproporphyrinogen III. However, cells expressing HCV core protein excreted larger amounts of coproporphyrin III compared with control cells. Because the extracellular content of coproporphyrin III did not change in parallel with the intracellular content (Figs 1 and 3), active export but not passive diffusion is likely involved. The decrease in intracellular coproporphyrin I in HCV core protein expressing cells was observed (Fig 1). Because of the decrease only in intracellular content, active transport system for coproporphyrin I might be responsible for this change. However, this decrease was not observed in another cell type (S2 Fig). Thus, it is unclear whether the decrease in intracellular coproporphyrin I is due to the HCV core protein expression or not. Abnormalities in porphyrin metabolism are detectable in the plasma and urine of patients with HCV infection, even those with no clinical manifestations of PCT. For example, Marttinelli et al. reported abnormalities in porphyrin metabolism in HCV-positive patients, as evidenced by increased levels of uroporphyrin in urine [17]. Additionally, Esmat et al. also reported a significantly higher prevalence of photosensitivity related to elevated porphyrins in asymptomatic cases of HCV infection [18]. Notably, Cribier et al. observed a significant increase in the urinary excretion of coproporphyrin in patients infected with HCV [19]. The results in this study may explain the altered urinary porphyrin excretion pattern, especially coproporphynuria, seen in HCV carriers who lack clinical evidence of PCT. Because urinary porphyrin levels correlate with total plasma porphyrin levels [20, 21], an increase in coproporphyrin III excretion by hepatocytes may lead to an accumulation of coproporphyrins in the plasma that then spills over into the urine.

To further explore the molecular basis of our observations, we studied the mRNA expression of enzymes and transporters involved in heme synthesis (Fig 4). In HCV core protein-expressing cells, increased UROD mRNA expression and decreased CPOX mRNA expression were observed, both with and without ALA treatment. These results may explain the change in intracellular porphyrin metabolism that leads to the excess production and excretion of coproporphyrin III observed in HCV core protein-expressing cells. Because UROD downregulation is known to be involved in PCT pathogenesis, these results suggest that the effect of HCV core protein expression does not entirely account for the features of PCT. Brudieux et al. reported that there was no correlation between hepatic porphyrin concentrations and hepatic UROD activity in HCV-infected patients who did not exhibit PCT [22]. These observations suggest that neither HCV infection nor HCV core protein directly causes the decrease in UROD activity. Several studies have suggested that HCV infection alone is insufficient to cause PCT; HCV infection may interact with other environmental and/or genetic factors to cause clinically overt PCT [19, 22].

Interestingly, the expression of ALAS1 protein, particularly in its mature form, was higher in HCV core protein-expressing cells than in control cells (Fig 6). Because ALAS1 is the rate-limiting enzyme for heme synthesis, our observations suggest that the porphyrin synthesis rate is likely higher in HCV core protein-expressing cells than in control cells. Increased ALAS1 activity in humans was reported to cause accumulations of dermato-toxic intermediates of the heme biosynthetic pathway [23]. Thus, the increased expression of mature ALAS1 is a possible factor that may account for the correlation between HCV infection and PCT.

Heme is known to strongly inhibit ALAS1 function via negative feedback [24]. Some studies have proposed a mechanism in which intracellular free heme binds to the pre-peptide of ALAS1 (pre-ALAS1) in the cytoplasm and prevents the precursor from translocating to mitochondria [25, 26]. Yoshino et al. reported that heme accelerates ALAS1 protein degradation and that its depletion leads to the accumulation of ALAS1 in rat liver mitochondria [27]. Based on the findings in these reports, the increased expression of mature ALAS1 is likely caused by the lower intracellular heme content in HCV core protein-expressing cells compared with controls (Fig 2).

Notably, we observed significantly higher levels of FLVCR1 and ABCG2 mRNA expression in HCV core protein-expressing cells compared with control cells (Fig 5). These transporters are involved in intracellular porphyrin and/or heme export. FLVCR1, a member of the major facilitator superfamily of transporter proteins, is a heme exporter that may play a critical role in erythropoiesis by protecting developing erythroid cells from heme toxicity [28]. Yang et al. demonstrated that FLVCR1 can export cyclic planar porphyrins, such as coproporphyrin and protoporphyrin IX [29]. ABCG2, a member of the ATP-binding cassette family of drug transporters, has also been observed to transport protoporphyrin IX in K562 and T24 cells and in mice [30]. As mentioned above, HCV core protein triggers abnormal porphyrin metabolism, which is evidenced in this study by increased coproporphyrin III excretion. These observations suggest the presence of a coproporphyrin III active transport system.

Because FLVCR1 and ABCG2 were both reported to transport coproporphyrin III, these transporters are candidates for involvement in the coproporphyrin III transport system observed in this study. In contrast to the observed mRNA expression levels, the ABCG2 protein expression was unchanged. This disparity between mRNA and protein expression suggests post-transcriptional and/or translational effects on the ABCG2 protein expression. The FLVCR1 protein expression was greatly increased by the expression of HCV core protein (Fig 6A and 6B). It was reported that cultured HepG2 cells are polarized and form pocket-like bile canaliculi [31, 32]. Thus, the increase in coproporphyrin III in the culture medium might be responsible for the basolateral efflux. Given that ABCG2 is localized in the canalicular (apical) membrane, FLVCR1, but not ABCG2, may play a major role in the abnormal excretion of coproporphyrin III observed in HCV core protein-expressing cells. Further studies are needed to fully investigate this possibility. The ABCB6 protein expression was slightly decreased, but not significantly, by the expression of HCV core protein as similar as the change in mRNA expression (Figs 5 and 6B). Considering a role of ABCB6 as a mitochondrial coproporphyrinogen III importer [15], the decreased ABCB6 expression may be responsible for the abnormal porphyrin metabolism in HCV core protein-expressing cells.

Iron also plays a central role in the development of PCT. Iron depletion through phlebotomy therapy is almost universally effective and produces a prompt remission of PCT [33]. Additionally, iron depletion therapy (in the form of dietary iron restriction and/or phlebotomy) can also improve hepatic inflammation and lower serum aminotransferase activity in HCV patients [34]. These observations indicate that iron plays a critical role in the pathologies of both disorders. Iron loading and increased oxidative stress lead to UROD inhibition and the oxidation of porphyrinogens to porphyrins in the liver [35, 36]. Previous work from our group demonstrated that HCV core protein induces mitochondrial iron accumulation and increased levels of ROS generation in HCV core protein-expressing cells and transgenic mice [11]. Together, the observations in that work and the present study suggest that the synergistic effect of iron overload and abnormal porphyrin metabolism may cause an increase in free iron that results from the decrease in heme synthesis. This increase in free iron might be an additional factor that plays a role in the pathogenesis of PCT.

In conclusion, this study indicates that HCV core protein decreases intracellular levels of protoporphyrin IX and heme and increases the excretion of coproporphyroin III in human hepatocellular carcinoma cells. These observations suggest that HCV infection triggers abnormal porphyrin metabolism in hepatocytes. Although the precise mechanism of how HCV core protein modulate the mRNA and protein expression in heme synthesis pathway is still unclear, the change in porphyrin metabolism that is induced by HCV core protein could at least partially account for the susceptibility to PCT development and the coproporphynuria seen in HCV carriers who lack clinical evidence of PCT. Our findings provide a greater understanding of HCV infection as a trigger of PCT pathogenesis and offer novel insights into the effect of HCV infection on hepatic heme biosynthesis and, more broadly, into the pathology of porphyrias.

## Supporting information

S1 Table Primer sets used for real-time PCR. (DOCX)

S1 Fig The effect of ALA treatment on cell viability. Hepswx and Hep39 cells were exposed to different concentrations of ALA for 24 h. At the end of the incubation period, the number of living cells were counted using Cell Counting Kit-8 as described in S1 Supplemental materials and methods. Data are presented as the mean ± SE of triplicate determinations.
(TIF)

S2 Fig The effect of HCV core protein on porphyrin accumulation and excretion in HCV core protein-expressing Huh-7 cells. A) Intracellular porphyrin accumulation in HCV core protein-expressing Huh-7 (Core) and vector control cells (Control). Cells were seeded into six-well culture plates at a density of 2.0 × 10^5^ cells/cm^2^; after 24 h, the cells were incubated with the 0.5 mM of ALA for 3 days, and the intracellular porphyrins were extracted as described in Materials and Methods. B) Porphyrin excretion into media from HCV core protein-expressing Huh-7 and vector control cells. Cells were seeded into six-well culture plates at a density of 2.0 × 10^5^ cells/cm^2^; after 24 h, the cells were incubated with the 0.5 mM of ALA for 3 days. Porphyrins in the medium were extracted as described in the Materials and methods. Column represents the mean (n = 2). Open circle represents the individual value.
(TIF)

S1 Supplemental materials and methods (PDF)
