# Supplementary material for: Hepatitis C virus core protein triggers abnormal porphyrin metabolism in human hepatocellular carcinoma cells
Source: PLoS One. 2018 Jun 1;13(6):e0198345. doi: 10.1371/journal.pone.0198345 (PMC5983478; doi:10.1371/journal.pone.0198345)
Supplement: S1 Table — (DOCX) [file pone.0198345.s001.docx]

Table 1. Primer sets used for real-time PCR
