# Supplementary figures and images for: Hepatitis C virus core protein triggers abnormal porphyrin metabolism in human hepatocellular carcinoma cells
Source: PLoS One. 2018 Jun 1;13(6):e0198345. doi: 10.1371/journal.pone.0198345 (PMC5983478; doi:10.1371/journal.pone.0198345)

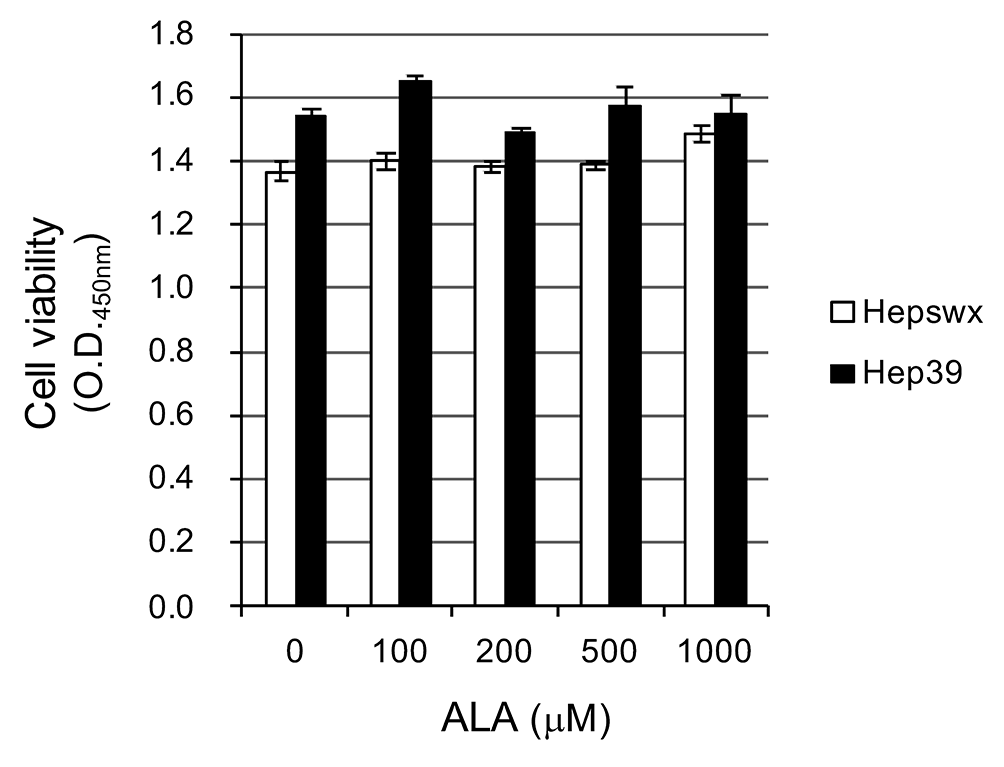

Supplement: S1 Fig — Hepswx and Hep39 cells were exposed to different concentrations of ALA for 24 h. At the end of the incubation period, the number of living cells were counted using Cell Counting Kit-8 as described in S1 Supplemental materials and methods. Data are presented as the mean ± SE of triplicate determinations. (TIF) [file pone.0198345.s002.tif]

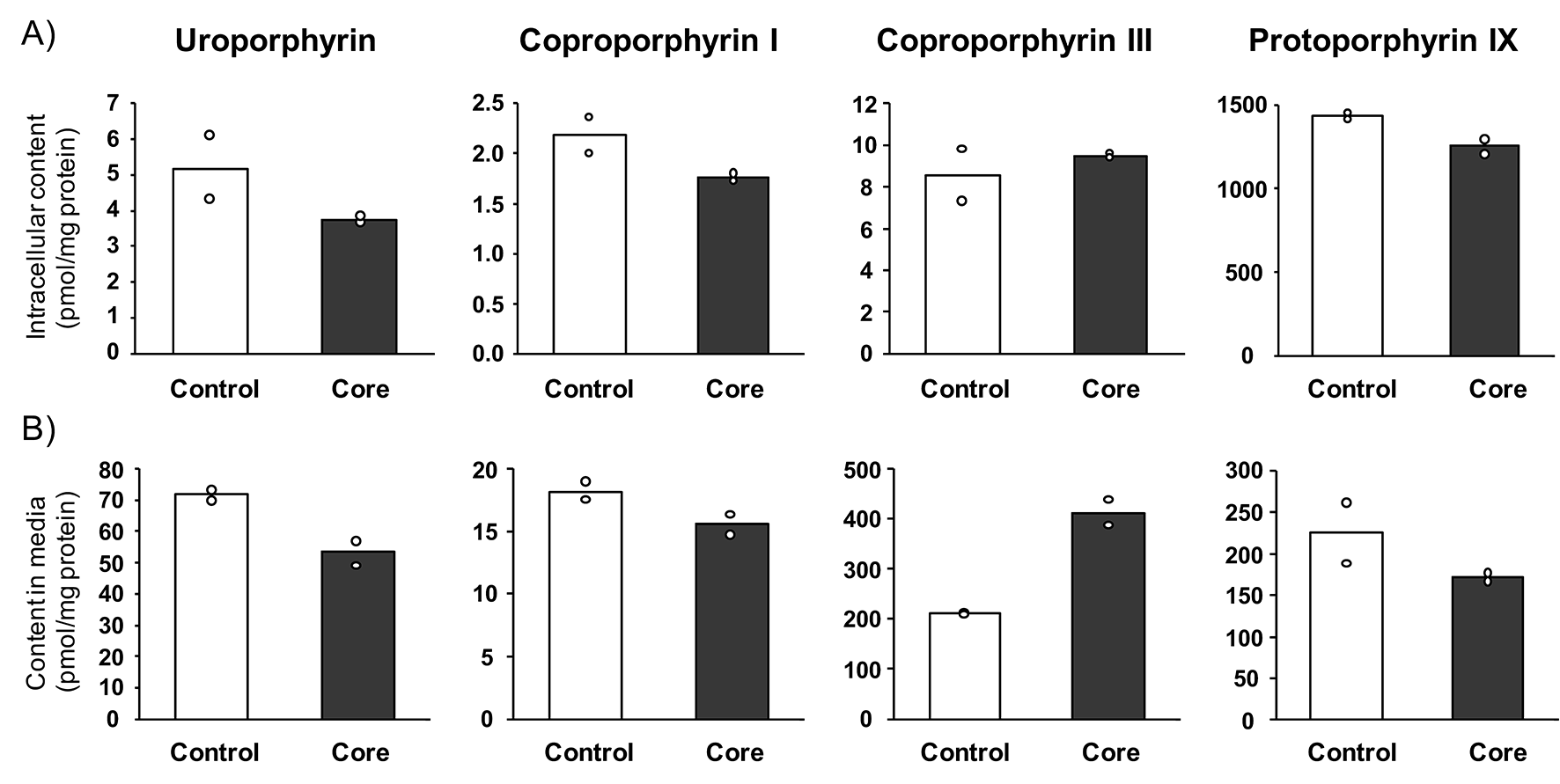

Supplement: S2 Fig — A) Intracellular porphyrin accumulation in HCV core protein-expressing Huh-7 (Core) and vector control cells (Control). Cells were seeded into six-well culture plates at a density of 2.0 × 10^5^ cells/cm2; after 24 h, the cells were incubated with the 0.5 mM of ALA for 3 days, and the intracellular porphyrins were extracted as described in Materials and Methods. B) Porphyrin excretion into media from HCV core protein-expressing Huh-7 and vector control cells. Cells were seeded into six-well culture plates at a density of 2.0 × 10^5^ cells/cm2; after 24 h, the cells were incubated with the 0.5 mM of ALA for 3 days. Porphyrins in the medium were extracted as described in the Materials and methods. Column represents the mean (n = 2). Open circle represents the individual value. (TIF) [file pone.0198345.s003.tif]
