# Supplementary material for: Hepatitis C virus core protein triggers abnormal porphyrin metabolism in human hepatocellular carcinoma cells
Source: PLoS One. 2018 Jun 1;13(6):e0198345. doi: 10.1371/journal.pone.0198345 (PMC5983478; doi:10.1371/journal.pone.0198345)
Supplement: S1 Supplemental materials and methods — (PDF) [file pone.0198345.s004.pdf]

Supplemental Materials and Methods

Establishment of Huh-7 cells stably expressing HCV core protein.

Huh-7 human hepatoma cells were obtained from RIKEN cell bank (Ibaraki, Japan). HCV core protein expression plasmid obtained from Addgene (CMV-FLAG-Core-R, Addgene plasmid 24480). Control vector plasmid was obtained by ligating the Hind III digested fragment of CMV-FLAG-Core-R, which did not contain HCV core cDNA. Huh-7 cells were seeded into 10-cm dishes at a density of 1.0 x 10^4 cells/cm^2, cultured for 12 h, and transfected with the constructed plasmids by lipofection method using a FuGENE HD Transfection Reagent. At 24 h after the initiation of transfection, the cells were selected in cell culture medium containing G418 (1.0 mg/ml) to obtain stable transfectants. The G418-resistant population was then maintained in the presence of G418 (0.5 mg/ml).

Assessment of cell viability

Cell viability was assessed by WST assay using a Cell Counting kit-8 (Dojindo Laboratories, Kumamoto, Japan) following the manufacturer’s instructions. Hepswx and Hep39 cells were seeded into 96 well culture plates at a density of 2.0×10^5 cells/cm^2 and, after 24 h, were exposed to various concentrations of ALA (0, 100, 200, 500, 1000 μM) for 24h. Then the number of viable cells was evaluated by the optical absorbance at wavelength 450 nm. The minimum cell viability was measured in Triton X-100 treated cells and subtracted from each sample as back ground.
